# More Caution Needed for Patients Recovered From COVID-19

**DOI:** 10.3389/fpubh.2020.562418

**Published:** 2020-11-02

**Authors:** Junguo Zhang, Hongying Qu, Cheng Li, Ziyi Li, Guanming Li, Junzhang Tian, Guowei Li

**Affiliations:** ^1^Center for Clinical Epidemiology and Methodology (CCEM), Guangdong Second Provincial General Hospital, Guangzhou, China; ^2^Department of Pulmonary and Critical Care Medicine, Guangdong Second Provincial General Hospital, Guangzhou, China; ^3^Guangdong Traditional Medical and Sports Injury Rehabilitation Research Institute, Guangdong Second Provincial General Hospital, Guangzhou, China; ^4^Department of Health Research Methods, Evidence, and Impact (HEI), McMaster University, Hamilton, ON, Canada

**Keywords:** COVID-19, SARS-CoV-2, recovered patients, re-detectable positive, countermeasures

## Introduction

Since December 2019, the COVID-19 (Coronavirus Disease, 2019) pandemic has caused ~35 million confirmed cases including over 1,000,000 deaths worldwide, and currently remains out of control in most countries (1). China is trying to bring COVID-19 under control by multiple drastic social and economic restrictions in combination with effective healthcare provision. As of Oct 7, 2020, among the 91,188 confirmed cases in China, only 386 cases remained hospitalized while there were 86,056 cases discharged after full recovery (1). The positive trend in China demonstrated a promising combat against the COVID-19 outbreak; however extreme caution is still needed given that the SARS-CoV-2 (Severe Acute Respiratory Syndrome Coronavirus 2) remains largely unknown to us. For instance, a recent study revealed that four recovered patients from COVID-19 showed positive quantitative RT-PCR (reverse-transcriptase–polymerase-chain-reaction) test results again after discharged from hospital (2). Likewise, data from Guangdong Province, China indicated that ~14% of the discharged patients would be tested positive for SARS-CoV-2 again (3). On April 10, the Korea Centers for Disease Control and Prevention also reported that more than 90 recovered patients showed recurrent positive test results for SARS-CoV-2 (4). These findings from recovered patients with re-detectable SARS-CoV-2 therefore raise a significant public health concern about whether they could spread SARS-CoV-2 to others again. To explore this public health concern, we systematically searched PubMed (up to Sep 9, 2020) to summarize the available evidence from studies that documented the recovered patients with re-detectable SARS-CoV-2, using the search terms (“novel coronavirus” OR “SARS-CoV-2” OR “COVID-19”) AND (“recovered” OR “discharged”) AND (“positive” OR “re-detectable”) with no language or time restrictions. Of 383 potentially relevant records, 36 eligible studies, independently reviewed by two investigators (JZ and GuoL), were included for analyses. For the purpose of prompt and easy intake, we also summarized the progress, symptoms, infectivity, potential reasons, and treatments of re-detectable positive patients in Table 1.

**Table 1:** Summary of the progress, symptoms, infectivity, potential reasons, and treatments of re-detectable positive patients.

Progress	Initial	In February 2020, four recovered patients from COVID-19 showed positive quantitative RT-PCR test result again.
	Developing	On April 10 2020, recurrent positive test results for SARS-CoV-2 were found in the Korea. Data showed nearly 14% of the discharged patients would be tested positive against.
Potential reasons	Virology	Viral residue, intermittent viral release, and periodic changes of virus replication.
	Detection of specimens	Low quality of throat swabs, different RT-PCR tests kits with imperfect accuracy, inadequate sampling and laboratory practices.
	Patients' condition	Older than 50 years and above, had comorbidities, received glucocorticoid therapy, longer hospital stay, lymphopenia, severe conditions, and lower immune
Symptoms	No symptoms or only mild symptoms.	
Infectivity	No reported and no conclusive evidence.	
Treatments	1. A combination of stool and different respiratory samples. 2. Employing the test of IgM-IgG antibodies. 3. Continual physical distancing or quarantines, use of respirator in public, close monitoring, and multiple laboratory tests for long-term follow-up after discharge.	

## Potential Reasons

Several possible reasons including virology, specimen detection and patients' condition, may help explain why the recovered patients with COVID-19 became retest positive for SARS-CoV-2.

### Virology

Currently nucleic acid detection represents the most widely used test to confirm SARS-CoV-2 infection. Following Guidelines for the Diagnosis and Treatment of Novel Coronavirus (COVID-19) Infection by the National Health Commission (Trial Version 7), 2 consecutive negative RT-PCR test results of respiratory specimens, mostly from throat swabs, are one key criterion for discharging patients with COVID-19 (5). However, the duration of SARS-CoV-2 RNA shedding has not been well-characterized (6). Viral RNA was detectable in different specimens, including throat swabs, stool, and urine, in patients for average 20 days (range, 8–37 days) after disease onset (7). Furthermore, recently studies have showed that higher viral loads are found in the nose and lower respiratory specimen than in the throat (8, 9); and the clearance of viral RNA in patients' stool is delayed compared to respiratory tract (10). A recent study with 98 patients showed that over half of stool samples of patients remained positive for SARS-CoV-2 for a mean of 11.2 days after respiratory tract samples became negative (11). The residuals and distribution of virus could thus be another possible reason for recovered patients' recurrence of positive viral test results.

### Detection of Specimens

Several aspects including decreased viral loads in patients due to their improving conditions, low quality of throat swabs, different RT-PCR tests kits with imperfect accuracy, inadequate sampling, and laboratory practices may yield variation of duration of viral shedding and false negative results and therefore inaccurate diagnoses of recovery. While widespread use of bronchoalveolar lavage fluid specimen test to detect SARS-CoV-2 is infeasible and impractical to help determine whether patients can be discharged, a combination of stool and different respiratory samples (especially from lower respiratory tract) may serve as a better tool to reduce the false negatives and recovered patients' recurrence.

### Patients' Condition

Once infected with SARS-CoV-2, the conditions of patients who were older than 50 years and above, had comorbidities or received glucocorticoid therapy might be more severe (7). SARS-CoV-2 in patients with severe COVID-19 had a longer duration of viral shedding and even could be detected until death (2, 7). Ultimately, longer hospital stay and lymphopenia due to severe conditions and lower immune function would more likely to result in retest positive of recovered patients (12).

## The Symptoms and Immunity of Recovered Patients

No obvious symptoms were reported in the recovered patients regardless of their RT-PCR test results after discharged from hospital. Similar to SARS-CoV-1 infection (13), virus-specific immunoglobulin M (IgM), immunoglobulin G (IgG), and neutralizing IgG antibodies were detected in most recovered patients between 7 and 14 days after the onset of symptoms, and antibody titers persisted for weeks following virus clearance (6). A recent study of SARS-CoV-2 infection in rhesus macaques indicated neutralizing antibodies against SARS-CoV-2 might offer protection to reinfection during early recovery days (28 days) (14). However, some study revealed that in a high proportion of recovered patients, IgG levels, and neutralizing antibodies started to decrease within 2–3 months after infection (15, 16). If patients' RT-PCR test was positive after recovery or immune systems become weak or declined, there remain potential risks of a relapse of symptomatic COVID-19 (17, 18). The IgM and IgG antibodies that were significantly and positively related to disease severity, would be produced gradually in the infected cases with COVID-19, which could help evaluate the stage of infection in the body (19). Therefore, employing the test of IgM-IgG antibodies as an add-on may be used to lower the risk of false negatives before a decision of hospital discharge was made. The test of IgM-IgG antibodies may also aid in the evaluation of whether recovered patients would require further close clinical attention after the recurrent positive RT-PCR test results.

## Infectivity and Treatment

Nevertheless, based on the literature there is no clear evidence showing that the SARS-CoV-2 found in patients recovered from COVID-19 is transmissible. Notably, detection of viral RNA does not necessarily indicate that infectious virus is present in specimens (16). A virological analysis of nine infected cases indicated no isolates of live virus after day 8 of symptoms onset, regardless of their ongoing high viral loads (20). Furthermore, one study found that only a low level of fragment genome could be detected in the recovered patients, implying that they would hardly spread the coronavirus again (21).

However, more caution is required because some other infective viruses have been known to persist for longer periods of time. For instance, infective Ebola virus could persists in semen for several months after two consecutive negative tests in blood samples (22). If human-to-human transmissions of SARS-CoV-2 were confirmed in patients recovered from COVID-19, the prevention and control would become significantly challenging and COVID-19 “immunity passports” would be challenged. While more high-quality evidence is urgently needed to determine the potential propagation in recovered patients with positive viral test results, continual physical distancing or quarantines, use of respirator in public, close monitoring, and multiple laboratory tests for long-term follow-up after discharge would be important for the current combat of COVID-19.

## Concluding Remarks

Although the phenomenon of discharged patients testing positive again for SARS-CoV-2 RNA was reported in some counties (e.g., Korea, Italy, and Russia), the studies were mostly documented from China. It is possible that these results from the strict and vigilant post-discharge monitoring policies in place in China (5). According to current reports, recovered patients with re-detectable SARS-CoV-2 account for a certain proportion of recovered patients. The underlying mechanism and infectivity of this population remains elusive and the evidence is only now starting to emerge. Considering the significance of this ongoing global public health emergency, we should take more caution needed for patients recovered from COVID-19 and perform more and urgent investigations of recovery cases to contain the epidemic.

## Author Contributions

JZ, JT, and GuoL: conception and design. JZ, HQ, CL, and GuoL: acquisition of data. JZ, ZL, JT, and GuoL: drafting the article. JZ, HQ, CL, ZL, GuaL, JT, and GuoL: revising it for intellectual content and final approval of the completed article. All authors: contributed to the article and approved the submitted version.

## Conflict of Interest

The authors declare that the research was conducted in the absence of any commercial or financial relationships that could be construed as a potential conflict of interest.
